# Raman Lasing and Transverse Mode Selection in a Multimode Graded-Index Fiber with a Thin-Film Mirror on Its End Face

**DOI:** 10.3390/mi15080940

**Published:** 2024-07-24

**Authors:** Alexey G. Kuznetsov, Vadim S. Terentyev, Victor A. Simonov, Hiba A. Rizk, Ilya N. Nemov, Kirill A. Bronnikov, Alexander V. Dostovalov, Sergey A. Babin

**Affiliations:** 1 Institute of Automation and Electrometry SB RAS, Novosibirsk 630090, Russia; 2 Department of Physics, Novosibirsk State University, Novosibirsk 630090, Russia; 3 Faculty of Physics, ITMO University, Saint-Petersburg 197101, Russia

**Keywords:** fiber laser, Raman laser, multimode, graded-index fiber, diode pumped, thin-film mirror, mode selection

## Abstract

Multimode fibers are attractive for high-power lasers if transverse modes are efficiently controlled. Here, a dielectric thin-film mirror (R~20%) is micro-fabricated on the central area of the end face of a 1 km multimode 100/140 µm graded-index fiber and tested as the output mirror of a Raman laser with highly multimode (M^2^~34) 940 nm diode pumping. In the cavity with highly reflective input FBG, Raman lasing of the Stokes wave at 976 nm starts at the threshold pump power of ~80 W. Mode-selective properties of mirrors with various diameters were tested experimentally and compared with calculations in COMSOL, with the optimum diameter found to be around 12 µm. The measured Raman laser output beam at 976 nm has a quality factor of M^2^~2 near the threshold, which confirms a rather good selection of the fundamental transverse mode. The power scaling capabilities, together with a more detailed characterization of the output beam’s spatial profile, spectrum, and their stability, are performed. An approximately 35 W output power with an approximately 60% slope efficiency and a narrow spectrum has been demonstrated at the expense of a slight worsening of beam quality to M^2^~3 without any sign of mirror degradation at the achieved intensity of >30 MW/cm^2^. Further power scaling of such lasers as well as the application of the proposed technique in high-power fiber lasers are discussed.

## 1. Introduction

It is generally accepted that the recent development of laser devices is mainly focused on their integrability into opto-electronic (photonic) circuits in the case of low-power chip-scale lasers [1] and on the design of robust diode-pumped high-power laser schemes with all-solid-state or all-fiber performance where laser cavity optics and pump elements are integrated into a single unit free of adjustable discrete parts [2]. For both of these extremes, hybrid schemes consisting of fiber and integrated optical elements produced with the use of various laser micro-fabrication technologies have been actively developed recently [3].

It is known that the most powerful fiber lasers based on Yb-doped active fibers have sufficient drawbacks, such as transverse mode instability and photo-darkening [2,4]. That is why high-power Raman lasing in passive fibers (which may eliminate these drawbacks) has been under active investigation in the last decade, with a focus on various designs of the fiber itself and cavity and pump coupling schemes [5]. In this direction, graded-index (GRIN) fibers in which multimode pump radiation may be directly coupled to the multimode fiber core are treated as one of the prospective solutions. It has been observed in various experiments that GRIN fibers provide efficient Raman conversion of multimode laser radiation into a Stokes beam with improved beam quality and brightness, which is known as the Raman beam cleanup effect [6]. The use of chip and reliable high-power multimode laser diodes (LDs) for pumping enables the development of new-type fiber lasers, namely, directly LD-pumped Raman fiber lasers (RFLs); see [7,8] for a review. Additional beam quality improvement is achieved in the Raman laser cavity consisting of fiber Bragg gratings (FBGs) inscribed in the GRIN fiber core, which also resulted in the development of stable and robust all-fiber integrated performance for such lasers [9].

With the development of all-fiber LD-pumped RFLs, impressive results have already been achieved in generating relatively high power radiation in the short-wavelength domain (950–980 nm) [7,9] not previously available neither in Yb-doped fiber lasers (YDFLs) nor in conventional RFLs based on single-mode passive fibers pumped by YDFLs [10]. The use in the laser cavity of output couplers based on a FBG inscribed by femtosecond (fs) pulses in the near-axis area of the GRIN fiber core, maximally overlapping with the fundamental mode, made it possible to maximize the effect of beam cleaning for the generated Stokes radiation. For example, such an all-fiber cavity consisting of highly reflective UV-inscribed FBG and fs-inscribed output FBG in a 1 km passive GRIN fiber of ~100 µm core diameter directly pumped by highly multimode LDs with beam quality parameter M^2^ ≈ 34 at 940 nm allows for the generation of a high-quality (M^2^ ≈ 2) Stokes beam at 976 nm with an output power of ~50 W [11]. In spite of the relatively large threshold pump power (~120 W), this experiment has demonstrated a record pump-to-Stokes brightness enhancement factor of about 73 in RFLs of this type. In such RFLs, it is also possible to explore random distributed feedback based on Rayleigh backscattering on natural refractive-index fluctuations in GRIN fibers, but such cavity feedback is rather weak, requiring multimode pumping at kW level, which was provided by combined output from several YDFLs [12].

Here, we study an alternative opportunity of integrated design for multimode RFL using, instead of internal Rayleigh backscattering or fs-inscribed FBG in-fiber output coupler, an output dielectric mirror microfabricated on the central area of the GRIN fiber end face. After femtosecond laser exposing the photoresist coated fiber end face and subsequent development process thus forming a hole, the output mirror with reflection R~20% (one TiO_2_ layer) was deposited by magnetron sputtering on the central part of the GRIN fiber end face so that it has transverse mode selective properties. Together with highly reflective (R > 90%) FBG UV-inscribed in the core at the input end of the GRIN fiber, where highly multimode (M^2^~34) LD pump radiation is coupled to the fiber, it forms a linear cavity, providing Raman lasing of the Stokes wave at 976 nm. In such a cavity, it appears possible to significantly reduce the threshold pump power (<80 W). The output beam quality factor measured near the threshold amounts to a near-diffraction-limited one (M^2^~2). The power scaling capabilities and transverse mode selection properties of the micro-fabricated mirror, together with a more detailed characterization of the output beam (spectrum, spatial profile, and their stability), have also been performed at increased pump power. The obtained characteristics are compared with those obtained in the same scheme with ~4% Fresnel reflection from the mirror-free fiber end face.

The potential of using such a micro-fabricated dielectric output mirror in the scheme of high-power LD-pumped multimode graded-index fiber Raman lasers is discussed, along with possible practical applications of this type of laser.

## 2. Materials and Methods

First, we developed a technology to micro-fabricate an output cavity mirror on the multimode fiber end face. There exist a number of works devoted to the fabrication of various structures at the end face of multimode fibers, in particular when it is necessary to perform spatial amplitude-phase filtering, or frequency filtering of radiation [13,14].

In our case, such an element is necessary for the task of selecting fundamental transverse mode in a multimode fiber Raman laser at a relatively high reflection coefficient that ensures lower threshold Raman lasing in multimode LD-pumped GRIN fiber than that obtained with fs-inscribed output FBG with an optimal (for mode selection) reflection coefficient of ~4% [7,11]. For this purpose, one should primarily develop a method for forming a dielectric mirror with a given spatial distribution in the cross section of a multimode fiber.

The developed technology includes several stages: preparation of the fiber end face, coating it with positive photoresist, exposure to femtosecond radiation, thus creating a mask, photoresist development, deposition of a thin-film coating, and cleaning the photoresist mask. In fact, this technology is analogous to standard photolithography technology presented in the literature; see, e.g., [15], where fabrication of sub-micron scale functional optical microstructures on the fiber end face by DMD-matrix-based lithography is demonstrated. However, there are significant aspects in our case, as described below. In addition, the micro-fabricated mirror should be stable in terms of radiation resistance since it is necessary to provide its long-term operation inside the cavity of a high-power multimode fiber laser.

**Figure 1:**
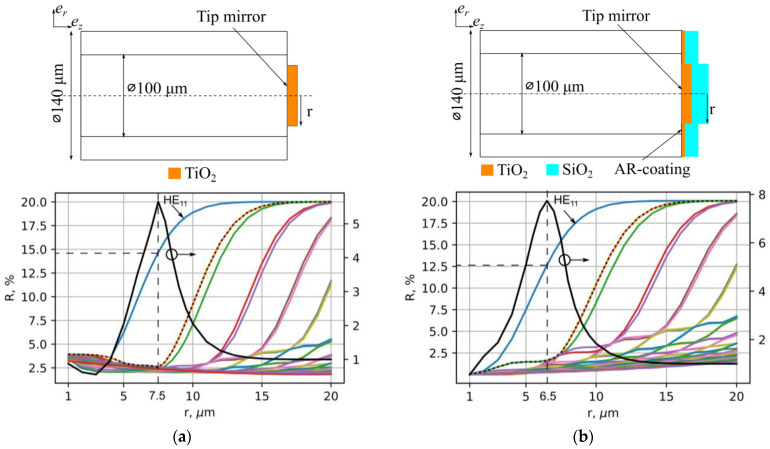
TiO_2_ thin-film mirror schematics (top) and overlap integrals of 45 modes depending on the radius of the mirror; the black line is the ratio of the overlap integrals of the first mode to the maximum of the others (dotted line): without (**a**) and with (**b**) an antireflection (AR) coating.

For transverse mode selection in a powerful multimode fiber laser, it is necessary to produce a reflector with a round shape located in the center of the fiber end face (tip), see Figure 1, capable of withstanding an intensity level of 10 MW/cm^2^. To excite predominantly the fundamental mode of the laser resonator, one should determine the optimal shape of the reflector (namely, its diameter) in terms of the minimum cavity loss for the fundamental mode relative to the higher-order modes. Using numerical modeling in COMSOL Multiphysics 6.0, the modes of the graded-index fiber with 100/140 µm core/cladding diameters were simulated, and their reflection from a TiO_2_ thin-film mirror was analyzed as a function of mirror diameter. The calculated reflection coefficient, which is the overlap integral of a chosen fiber mode with the distribution reflected from the mirror, is shown in Figure 1 for the first 45 radial modes with an angular index of 1. One can see that all the curves are similar, showing fast growth of the reflection coefficient with increasing mirror radius, which is then saturated at the chosen level (R = 20%). At the same time, the saturation point is shifted to larger mirror radii for higher-order modes so that there exists an optimal ratio of the fundamental mode (HE_11_) reflection coefficient to the maximal one for higher-order modes $R_{11}/max(R_{mn})$, shown by the black lines in Figure 1.

As the interference of partial reflection from the thin-film mirror and uncoated part of the fiber end face appears to be sufficient, the calculations were carried out for the cases both without (a) and with (b) a TiO_2_/SiO_2_ antireflection (AR) coating; see the upper inset in Figure 1b. However, such AR coating only slightly influences the optimal mirror diameter; it was estimated to be 14–16 µm (Figure 1a) and 12–14 µm (Figure 1b) in these two cases. We have also checked that accounting for the cross-terms only slightly changes this picture, resulting in a small shift of the optimum diameter within 1 µm to lower values, but its quantitative contribution depends on the concrete composition of the analyzed beam.

To fabricate the proposed thin-film mirrors experimentally, segments of GRIN fiber (Draka 100/140) prepared and glued to Canadian balsam in fiber ferrules were fixed in a special holder (similar to the technique used in [15]) and coated by centrifugation method with a layer of photoresist FPN-2506 up to 1 µm thick, diluted 3 to 1.5. The photoresist was exposed using a femtosecond laser setup with up to 6 W average power at a pulse repetition rate of 200 kHz, which was earlier used for the fabrication of anti-reflection microstructures on the nonlinear crystal surfaces [16]. In addition, the laser output pulses with a duration of 230 fs at a wavelength of 1026 nm were converted to the second harmonic at 513 nm for the sake of photopolymerization. The laser pulse energy level and scanning speed (average number of pulses in the focal spot of a high-NA objective) were varied to optimize the photopolymerization process.

**Figure 2:**
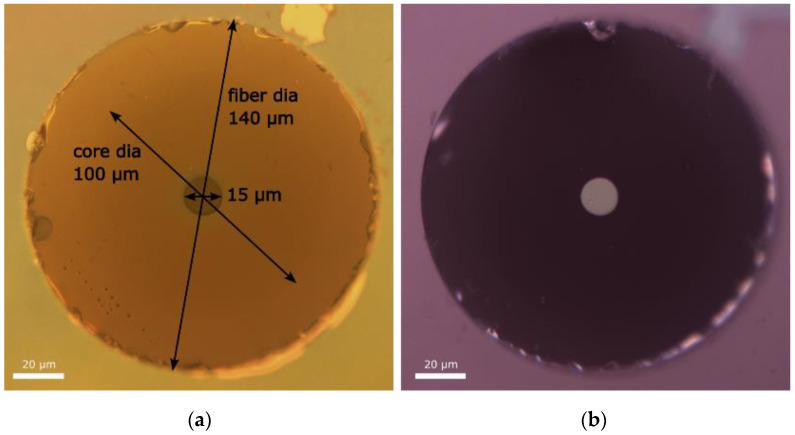
The microscope image of the end face of the Draka 100/140 GRIN fiber: (**a**) the developed photoresist mask; (**b**) the TiO_2_-film mirror with a diameter of 15 micron after cleaning.

After the photoresist developed, a mask was formed on the fiber end face with a hole in the center (Figure 2a). A single TiO_2_ layer with a thickness of about 60 nm (without AR coating) was deposited onto this mask by magnetron sputtering in vacuum with a reflection coefficient of around 20%. After deposition, the photoresist mask was removed using acetone (Figure 2b). In this way, several mirrors with diameters of 10, 12, and 15 µm were fabricated. For one of two 10 µm mirrors, another deposition of TiO_2_/SiO_2_ layers was performed in order to add AR coating. After the mirror fabrication, the fiber pieces were extracted from the ferrules by heating them up to the melting point of Canadian balsam. Then, the fibers were cleaned from the remnants of the balm, and the end face was finally cleaned with a special cloth for fiber cleaning.

**Figure 3:**
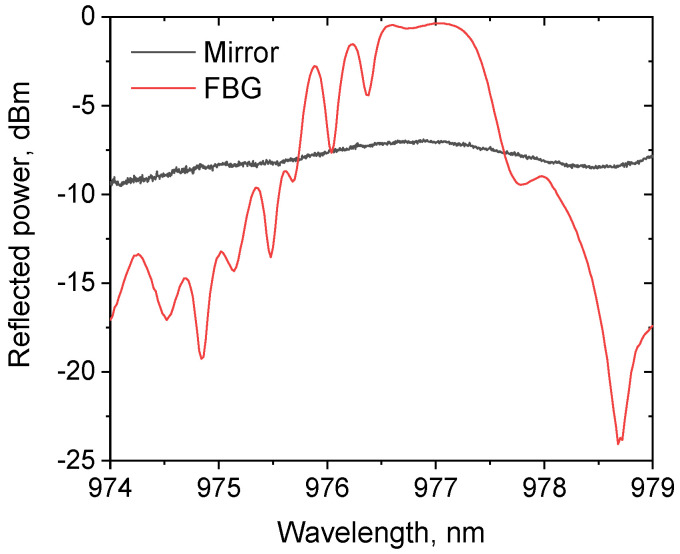
Reflectance spectra of the input FBG and output dielectric mirror with a diameter of 15 microns.

In Figure 3, the reflection spectra of the output thin-film mirror and of the FBG used as the highly reflective cavity mirror, which was inscribed by UV laser in the GRIN fiber core (see [7,9] for more details), are shown. A weak modulation present in the reflection spectrum of the thin-film mirror (which is sensitive to fiber bending) is associated with the intermode interference in the output fiber segment, although average reflection is nearly constant (checked in the 970–990 nm interval). At the same time, the highly reflective (R~90%) FBG has a resonant reflection profile with a main peak near 976.5 nm, which corresponds to the fundamental mode, whereas higher-order mode groups have corresponding resonances at the short-wavelength tail of the FBG reflection spectrum with a period of ~0.4 nm in the 100 µm core GRIN fiber (see [7,9] for more details). Their amplitudes decrease to −10–15 dB near 975 nm, which corresponds to resonances of around 5th order modes. So, it can be considered that in such a laser cavity, only the FBG is a spectrally selecting element, while the thin-film output mirror has a relatively uniform reflection spectrum in the spectral region under study, but it should have better transverse mode selective properties in the spatial domain, as illustrated in Figure 1.

**Figure 4:**
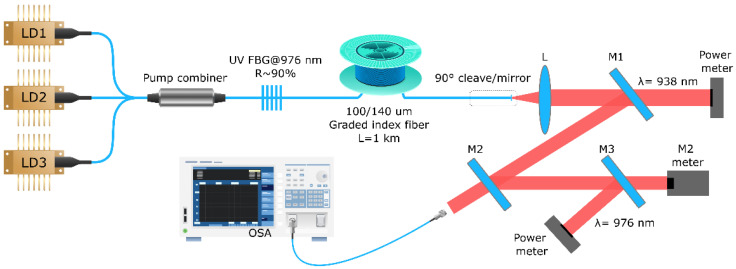
RFL cavity scheme: LD1, LD2, LD3—multimode laser diodes; UV FBG—high-reflection fiber Bragg grating inscribed by UV laser with corresponding Bragg wavelength; L—collimating lens; M1, M2, M3—dichroic mirrors; OSA—optical spectrum analyzer; Power meters and M^2^ meters are shown by black boxes.

At the next step, the micro-fabricated thin-film mirrors were tested as output couplers in a multimode Raman fiber laser. Figure 4 shows the cavity scheme of the RFL under the test. Pump radiation from three fiber-pigtailed high-power LDs operating at a wavelength of ~940 nm is added together by a 3 × 1 multimode fiber pump combiner. The input ports of the fused combiner are made of multimode step-index fiber with a 105-μm core, and the output port is made of a 100-μm-core Draka GRIN fiber with a numerical aperture of 0.29. It is spliced to the same 1 km-long 100-μm core GRIN fiber in which the Raman gain is provided by LD pumping. The linear cavity was formed by a highly reflective FBG (R~90%) and a thin-film mirror deposited on the output fiber end face (R~20%) described above. Also, to compare lasing regimes, the output mirror was replaced by a conventional fiber end cleaved at a straight angle, which provides ~4% Fresnel reflection without selection of transverse modes.

For the RFL characterization, we used dichroic bulk mirrors M1, M2, and M3 in order to separate pump radiation at 940 nm so that the first power meter measures residual pump power (transmitted through M1) while the Stokes radiation at 976 nm reflected by bulk mirrors M2 and M3 is registered by the second power meter. In addition, some residual radiation passed through M2 and M3 is used to measure the output spectrum and profile of the generated beam by an optical spectrum analyzer (OSA) and Thorlabs M^2^-measurement system, respectively. Using a camera, images of the intensity distribution for the pump and Stokes beams at the fiber output end face were obtained. To do this, the pump laser diodes were turned on below the lasing threshold and then in the regime high above the threshold when Stokes generation started in the fiber laser.

## 3. Results

**Figure 5:**
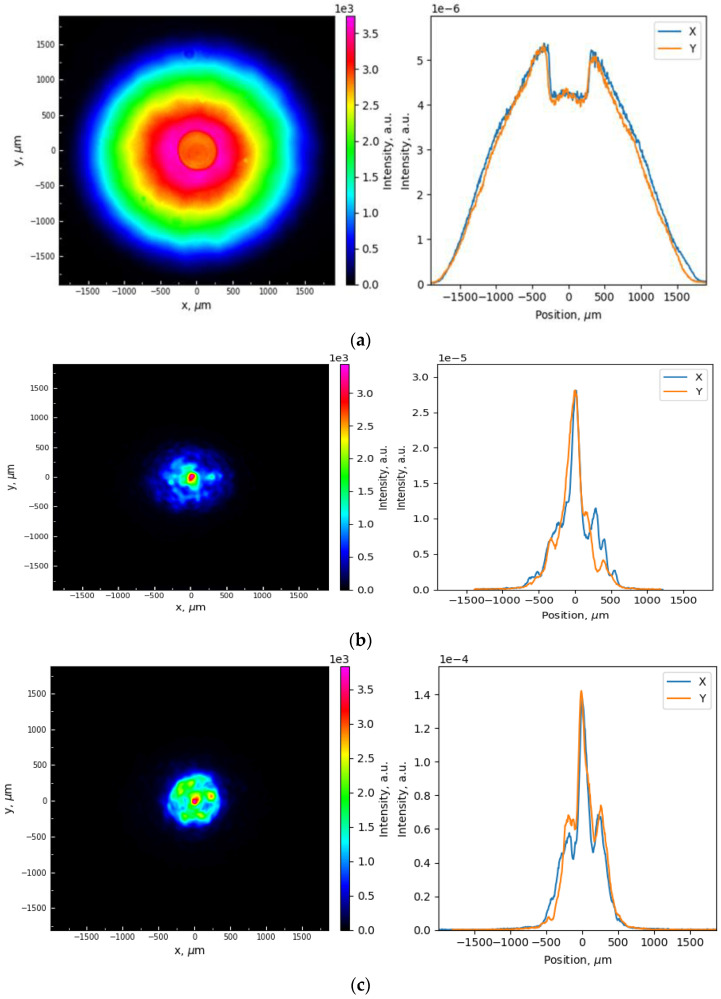
Intensity distribution of the output pump below the Raman threshold (**a**) and the generated Stokes beam at 4 W power (**b**) for a 15 µm mirror. Intensity distribution of the generated Stokes beam at 31.4 W output power for a 12 µm mirror (**c**).

Figure 5 shows the intensity profiles of the transmitted spontaneous emission of the pump diode before the Raman threshold (a) and the intensity of the Stokes beam above the threshold (b) measured in the plane of the fiber end face with a thin-film mirror of 15 µm diameter (shown in Figure 2b). The end-face image is focused into the camera with corresponding enlargement. The pump beam measured in this way represents an almost parabolic profile with a diameter corresponding to the GRIN fiber core (Figure 5b), in the center of which a circular region of the mirror and a corresponding ~20% dip in the radiation intensity profile are visible. When Stokes radiation starts to generate with increasing pump power, a narrow 976 nm beam is observed in the cross section of the mirror, as well as a speckled substrate slightly extended beyond the mirror region. The Stokes beam profile shown in Figure 5b has a power of 4 W, and its beam quality parameter is measured to be M^2^ ≈ 2.3 (slightly higher than M^2^ ≈ 2.0 measured at 1 W), which is good enough in spite of the significant contribution of speckled background. With increasing output power, the beam quality degrades sufficiently to M^2^ ≈ 3.8 at 18 W output power, which is worse than the best result for RFLs with fs-inscribed output FBG at 976 nm [11]. To improve the beam quality at high powers, we also tested thin-film mirrors of smaller diameters in the RFL cavity (Figure 4). The best beam quality was obtained for a 12 µm mirror, with which M^2^ ≈ 3.0 at 21 W is observed without significant changes in quality parameters at further power growth. the corresponding Stokes beam profile at 31.4 W output power is shown in Figure 5c. One can see less speckles and a more regular beam profile, with ~20% side spikes clearly seen in the X and Y cross sections, which correspond to the mirror edges. Similarly to the pump beam (Figure 5a), the mirror reduces the intensity of the central part of the Stokes beam, whereas its tails extended beyond the mirror diameter remain unchanged.

**Figure 6:**
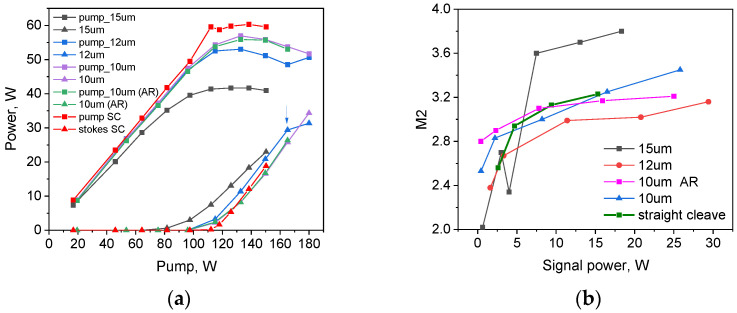
Laser output power (**a**) and beam quality (**b**) for different mirror radii in comparison with a straight cleave. In (**a**), residual pump power is also shown. The second Stokes threshold observed for a 12 µm mirror is shown by the arrow.

Output power and beam-quality characteristics of the multimode GRIN-fiber Raman laser in configurations with the ~20% end-face mirror of different diameters (15, 12, and 10 µm) are compared in Figure 6a,b, together with the data obtained with straight cleave (SC) instead of the mirror. The laser with ~4% SC Fresnel reflection has the highest threshold of ~120 W (Figure 6a), and above the threshold, the Stokes beam quality parameter is slightly growing from M^2^~3 at 5 W to M^2^~3.2 at 15 W. The laser with a 10 µm wavelength has a slightly lower threshold, but with increasing pumping, its output power and beam quality parameters tend to follow the characteristics obtained with mirror-free SC reflection: M^2^~3.2 (at 16 W) slightly increases with output power reaching 35 W at about 180 W pumping. Note that the laser with a 15 µm mirror has the lowest threshold (~80 W) and quite a good beam quality parameter at low output power (M^2^~2 at 1W), but already at >7.5 W output power, the beam quality degrades to M^2^ > 3.6, which is even worse than that for straight cleave. The laser with a 12 µm mirror has an intermediate threshold (~100 W) and the best characteristics well above the threshold: output power grows with the highest slope efficiency of about 60%, while beam quality remains the lowest up to the level of about 30 W at ~165 W pumping.

**Figure 7:**
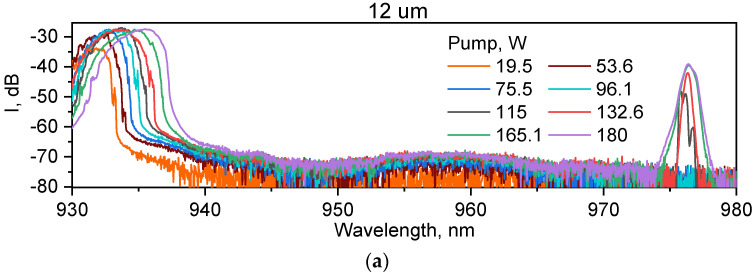

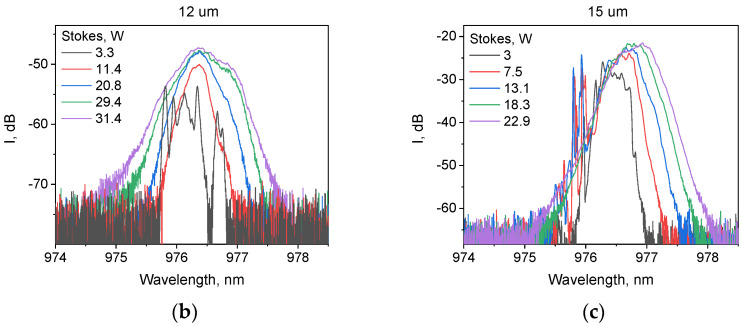
Output spectra of RFLs in configurations with a 12 µm mirror in the broad range (**a**) and the Stokes spectrum near 976 nm (**b**) and the Stokes spectrum near 976 nm with a 15 µm mirror (**c**) at different powers.

Note that for 12 µm mirror RFL, further power growth (to 31.4 W) is limited by starting the second-order Stokes generation at a power of 29.4 W, which additionally confirms the high quality of the first Stokes beam that plays a role of the pump beam for the second-order Raman lasing. The optical spectrum of the generated Stokes wave for RFLs with a 12 µm output mirror is shown together with the pump spectrum in Figure 7a, and the detailed Stokes spectrum near 976 nm is shown in Figure 7b. It is stable and relatively narrow (−3 dB width is <0.5 nm) up to the maximum power of 31.4 W; some instability of the Stokes spectrum is observed only near the threshold (at 3.3 W output power). For the 15 µm mirror (Figure 7c), the measured Stokes spectrum is unstable at almost all high-power domain (≥7.5 W), showing unstable spikes at the short-wavelength tail of the spectrum corresponding to higher-order mode resonances in the FBG spectrum (see Figure 3). Such unstable appearance of higher-order modes may also be responsible for excessive degradation of beam quality for RFLs a with a 15 µm mirror (see Figure 6a). For the 10 µm mirror, the spectrum is also less stable than that for the 12 µm mirror, with high-order mode spikes observed in the 2–16 W power range.

We have also tested a 10 µm mirror with an AR coating, as the coating shifts the optimal mirror diameter to smaller values (see Figure 1), but we did not succeed in the full elimination of the reflection from the peripheral area (the rest of the reflection is estimated at 1–2%). So, the RFL characteristics for the 10 µm mirror with AR coating are measured to be close to those for the 10 µm mirror without AR coating (see Figure 6); the only improvement is the better beam quality parameter at high powers (M^2^ ≈ 3.2), which is close to the best M^2^ values obtained for the 12 µm mirror.

**Figure 8:**
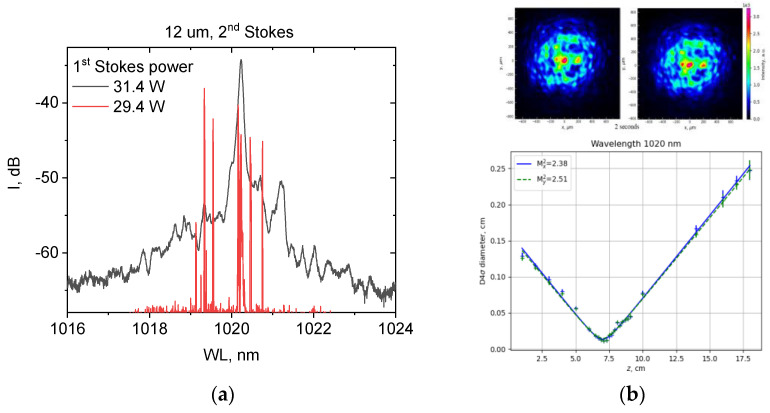
Second Stokes beam spectrum near the threshold (29.4 W of first Stokes power) and above the threshold (31.4 W of first Stokes power) (**a**); second Stokes beam shape measured in intervals of 2 s through additional filter and M^2^ measurements near the beam waist (**b**).

For multimode RFLs with a 12 µm mirror, we also characterized the second Stokes beam, namely its spectrum (Figure 8a) and shape (Figure 8b) with temporal resolution. Near the second Stokes threshold, corresponding to 29.4 W of the first Stokes power (serving as the Raman pump), the second Stokes laser spectrum represents narrow random spikes near the maximum of the Raman-amplified spontaneous emission peak centered at 1020 nm. At 31.4 W of the first Stokes power, the multi-peak spectrum collapses into a single stable peak (with a −3 dB width below 0.1 nm) well above the random lasing threshold. This behavior is typical for random Raman lasing, either in singlemode [17] or in multimode fibers [12,18]. In our case, the random lasing arises in the half-open in-fiber cavity, comprising the end-face 20% broadband mirror (also reflecting at 1020 nm) and random distributed feedback via Rayleigh backscattering in the multimode 1 km long GRIN fiber. The main second Stokes output in such a cavity is directed from the mirror to the pump end, given that the 976 nm UV FBG is nearly transparent at 1020 nm. The second Stokes output power measured from the mirror end amounts to 0.3 W, so the power in the opposite direction near the pump end is estimated to be >7 W, and its further increase is dangerous for pump LDs. The estimated second Stokes power is in good correspondence with the relative depletion of the first Stokes power and the increase in pump power in the last point (at 180 W pumping) for RFLs with a 12 µm mirror in Figure 6a. The second Stokes beam shape measured at the end face with mirror consists of a bright spot and speckled background that is stable in time (Figure 8b, top). At the same time, the corresponding M^2^ parameter extracted from D_4σ_ beam diameter measured as a function of coordinate along the focused beam is rather good, with M^2^ ≈ 2.4 (Figure 8b, bottom), at which point the beam shape in the waist becomes smoother. As a result of a high-quality second Stokes beam with relatively high power, the third-order Stokes amplified emission becomes visible near 1070 nm.

## 4. Discussion and Conclusions

Thus, we have successfully demonstrated the opportunity to use for mode selection in multimode LD-pumped 100 µm GRIN-fiber RFL of an output (R~20%) dielectric thin-film mirror micro-fabricated on the central area (10–15 µm) of the fiber end face. Together with highly reflective (R~90%) FBG UV-inscribed in the core at the input end of GRIN fiber where highly multimode (M^2^~34) broadband LD pump radiation around 940 nm is coupled to the fiber, it forms a linear cavity providing Raman lasing of low-order transverse modes Stokes beam at 976 nm above the relatively low threshold pump power (~80 W). The output beam quality factor measured near the threshold (in the 1–4 W output power range) is close to the diffraction limit, M^2^ ≈ 2–2.3, but it worsens sufficiently (M^2^ > 3.6) at the increase in generated power to ≥10 W with the mirror of relatively large diameter (15 µm). At the same time, the laser spectrum becomes less stable with spikes observed at its short-wavelength tail corresponding to higher-order transverse mode resonances in the FBG spectrum. Such beam quality and spectrum degradation with increasing power may be explained by the relatively large diameter of the 15 µm mirror (slightly larger than that for fundamental mode), at which higher-order modes start to generate and become unstable well above the threshold. Testing the mirror with a diameter (10 µm) sufficiently smaller than the fundamental mode has shown that it allows for Stokes beam generation with a beam quality parameter tending to the characteristics obtained with mirror-free 4% Fresnel reflection, M^2^ > 3.2 at >16 W, which value slightly increases with increasing power up to 35 W at about 180 W pumping. So, such a small mirror does not have so much of an effect in comparison with the Fresnel reflection from the peripheral uncoated part.

The best results in terms of Raman generation slope efficiency (about 60%), stable narrow spectrum (FWHM < 0.5 nm), and output beam with rather good mode selection (M^2^~3) in the high power domain have been obtained with a thin-film mirror of 12 µm diameter (slightly lower than that for fundamental mode and the optimal diameter estimated from COMSOL simulations). As a result, the thin-film output mirror micro-fabricated on the fiber end face provides comparable LD-pumped RFL output characteristics and brightness enhancement factor with those for in-fiber fs-inscribed output FBG, but at a sufficiently lower threshold pump power. The specifics of the thin-film mirror also consist of broadband reflection, which leads to partial back-reflection of the pump beam (low-order modes) that reduces the Raman threshold. However, it also limits the maximum power of the generated first Stokes wave by reducing the threshold for the second Stokes wave that starts to generate via random lasing in the half-open cavity formed by the thin-film mirror and the Rayleigh backscattering in GRIN fiber. At that, the generated second Stokes wave at 1020 nm has a high beam quality (M^2^~2.4) at backward power estimated as >7 W and a very narrow spectrum (FWHM < 0.1 nm) in spite of the speckled background present in the beam profile. At the same time, the radiation load on the mirror at maximum power is estimated to be >30 MW/cm^2^, without any sign of degradation.

It should be noted that we optimized the output thin-film mirror characteristics at the fixed GRIN fiber length of ~1 km, which was defined as optimal for the RFLs with an output coupler based on fs-inscribed FBG [7,9,11] at a maximum pump power of about 200 W. Here, the pump power is limited by nearly the same value (180 W), and the obtained slope efficiency of pump-to-Stokes conversion (~60%) corresponds to the linear losses in the 1 km GRIN fiber, similar to the RFL scheme with output fs-FBG. With the optimal mirror of 12 µm in diameter, the second Stokes threshold observed at 165 W manifests that the GRIN fiber length may be slightly shorter, thus shifting the second Stokes threshold to maximum pump power and increasing the maximum first Stokes power. However, this factor allows only slight improvement. A more significant improvement in slope efficiency (and absolute efficiency as well) with shortening of GRIN fiber by several times requires corresponding enhancement of coupled pump power level that may be possible by adding more laser diodes with less losses at their combining and coupling to the GRIN fiber, which represents a rather complicated technical task but is potentially possible.

Thus, the presented proof-of-principle experiment demonstrates high radiation resistance and good mode-selective properties of the thin-film mirror micro-fabricated on the multimode fiber end face. With further optimization of the dielectric mirror and anti-reflection coating (which may significantly improve the mode selection efficiency), it can result in the simple and robust performance of LD-pumped RFLs with power scalable to hundreds of Watts and more, which is important for extending its application range, including material processing, harmonics generation, laser displays, etc. The large spectral bandwidth of such mirrors in comparison with FBG also offers the opportunity for easy wavelength tuning of such lasers, for which it is enough to tune the HR FBG reflection wavelength. It may also be combined with efficient cascaded generation of multiple higher Stokes orders in a broad range with its use together with multiple highly reflective FBGs that may potentially offer one-octave tuning of LD-pumped RFLs, similar to conventional YDFL-pumped RFLs [19], but at sufficiently higher power obtained in simple and robust all-fiber performance with direct LD pumping.

## Data Availability

Data are available upon request.
